# Assessing cortical features in early stage ASD children

**DOI:** 10.3389/fpsyt.2023.1098265

**Published:** 2024-01-10

**Authors:** Antonio Napolitano, Silvia Guerrera, Martina Lucignani, Chiara Parrillo, Giulia Baldassari, Francesca Bottino, Giulia Moltoni, Maria Camilla Rossi Espagnet, Lorenzo Figà Talamanca, Giovanni Valeri, Stefano Vicari

**Affiliations:** ^1^Medical Physics Unit, Bambino Gesù Children’s Hospital, IRCCS, Rome, Italy; ^2^Neuroscience Department, Child Neuropsychiatric Unit, Bambino Gesù Children’s Hospital, IRCCS, Rome, Italy; ^3^Imaging Department, Neuroradiology Unit, Bambino Gesù Children’s Hospital, IRCCS, Rome, Italy; ^4^Department of Neuroradiology, NEMOS S. Andrea Hospital, University Sapienza, Rome, Italy; ^5^Child and Adolescent Neuropsychiatry Unit, Bambino Gesù Children’s Hospital, IRCCS, Rome, Italy; ^6^Department of Life Science and Public Health, Università Cattolica del Sacro Cuore, Rome, Italy

**Keywords:** autism, MRI, cortical thickness, local gyrification index, verbal ability

## Abstract

Autism Spectrum Disorder (ASD) is defined as a neurodevelopmental disorder largely investigated in the neurologic field. Recently, neuroimaging studies have been conducted in order to investigate cerebral morphologic alterations in ASD patients, demonstrating an atypical brain development before the clinical manifestations of the disorder. Cortical Thickness (CT) and Local Gyrification Index (LGI) distribution for ASD children were investigated in this study, with the aim to evaluate possible relationship between brain measures and individual characteristics (i.e., IQ and verbal ability). 3D T1-w sequences from 129 ASD and 58 age-matched Healthy Controls (HC) were acquired and processed in order to assess CT and LGI for each subject. Intergroup differences between ASD and HC were investigated, including analyses of 2 ASD subgroups, split according to patient verbal ability and IQ. When compared to HC, ASD showed increased CT and LGI within several brain areas, both as an overall group and as verbal ability an IQ subgroups. Moreover, when comparing language characteristics of the ASD subjects, those patients with verbal ability exhibit significant CT and LGI increase was found within the occipital lobe of right hemisphere. No significant results occurred when comparing ASD patients according to their IQ value. These results support the hypothesis of abnormal brain maturation in ASD since early childhood with differences among clinical subgroups suggesting different anatomical substrates underlying an aberrant connectivity.

## 1 Introduction

Autism Spectrum Disorder (ASD) is defined as a neurodevelopmental disorder characterized by the presence of persistent deficits in social communication and social interaction, restricted and repetitive patterns of behavior, interests or activities (1). By definition, the symptoms occur early and affect daily functioning. ASD affects 1 in 54 children in the United States, with a prevalence that is four times greater in boys than in girls (2). ASD is considered a neurodevelopmental disorder associated with neurologic changes with an onset in prenatal or postnatal life, modifying the typical pattern of child development (3). Although the etiology is considered multifactorial, genetic and environmental risk factors can both contribute to the development of the disorder. Recently, neuroimaging studies have been conducted in order to investigate cerebral morphologic alterations in ASD patients and demonstrated an atypical brain development before the clinical manifestations of the disease thus, suggesting a possible predisposing neuroanatomical prenatal condition (4). Recent studies have explored brain maturation in high-risk children, during the first six months of life, demonstrating an atypical development of sensory connectivity in children who will later develop ASD (5). Between 6–12 months of age, children who develop signs of ASD could show an increase of cortical surface, involving regions for auditory and visual processing, followed by a more global overgrowth within the following 24 months (6, 7). In children with ASD aged 2 to 4 years, cerebral volumes remain increased compared to those of children with neurotypical development (8), especially in brain areas correlated to social cognition, verbal abilities, and emotion regulation. Cerebral growth declines in school aged children and adolescents with a slow growth and with brain volumes of ASD similar to neurotypical children (7–11). Different cortical measures have been investigated, such as volume, surface area (SA) cortical thickness (CT) and local cortical gyrification (LGI) (12–17). Kohli and colleagues compared cortical morphology between individuals with ASD and neurotypical children, analyzing cerebral gyrification. Their results indicated that LGI measures of children with ASD increased in some cortical regions but decreased with older age than neurotypical children (14). Smith and colleagues investigated changes in CT and SA in children aged 2 to 9 years, finding a progressive reduction of CT in neurotypical children but not in children with ASD (18). Another study of older children and adolescents (8–15 years old) found a faster reduction in CT of ASD than neurotypical children, involving temporal and occipital brain areas (19). These results confirm conclusions of a previous study of Zielinski and colleagues, who performed a longitudinal study of cortical thickness in a large group of individuals with ASD with an age range of 3–39 years. The authors showed an increased cortical thickness in early childhood, followed by accelerated thinning into later childhood and adolescence involving frontal and later posterior brain areas; finally, a decelerated thinning in young adulthood (17). Based on literature evidences reporting brain maturation alteration in the first years of ASD patients, the purpose of our study was to investigate cortical features such as CT and LGI in a large sample of ASD children with respect to a healthy controls group. In particular, we investigated possible relationships between brain measures and individual characteristics such as intelligence quotient and verbal language in the ASD population studied.

## 2 Materials and methods

### 2.1 Subjects

This is an observational, cross-sectional, non-interventional, single-center study approved by the Institutional Ethical Committee. Written informed consent from a parent/guardian of each participant was obtained when filling out the questionnaire. Patients were enrolled in the study if fulfilling the following inclusion criteria: a) diagnosis of ASD according to the DSM-5 criteria and confirmed by Autism Diagnostic Observation Scale-2 (ADOS-2); b) age between 2 and 8 years. Exclusion criteria were: a) the presence of behavioral problems that did not allow testing; b) genetic abnormalities based on pathogenic findings from CGH microarray or syndromic autism. Additionally, a group of age-matched healthy controls (HC) was included. Inclusion criteria for HC were: a) age between 2 and 8 years and b) absence of neurological or psychiatric disorders, while exclusion criteria were: a) presence of MRI abnormalities; b) having a first-degree relative with ASD; c) history of premature birth or extremely low birth weight and d) history of special education services/early intervention before enrollment. All subjects underwent MRI examination that was reviewed in consensus by two pediatric neuroradiologists. Moreover, all ASD subjects were also evaluated with verbal cognitive (IQ) score. All neuropsychological tests were conducted by trained developmental psychiatrists and neuropsychologists.

### 2.2 Neurophsycological measures

The ADOS-2 is a semi-structured direct assessment of communication, social interaction, and play or imaginative use of materials for individuals with a suspected diagnosis of ASD. The ADOS-2 consists of five modules designed for children and adults with different language levels, ranging from nonverbal to verbally fluent. The ADOS-2 was administered and scored by licensed clinicians who have demonstrated clinical proficiency on the instrument. The calibrated severity score of each domain was also calculated and was used to endorse the diagnosis of ASD. Comparison scores (CS) were considered for the ADOS-2 analysis. Cognitive development was assessed by the nonverbal intelligence quotient (IQ) obtained from the Leiter International Performance Scale, Revised (Leiter-R) (20) or Third Edition Leiter-3 (21), or by the Griffiths Mental Development Scales-Extended Revised for age 2–8 (GMDS-ER 2–8) (22) and Griffith III. The Leiter-R and Leiter-3 offer a completely nonverbal measure of intelligence and evaluate the ability to reason by analogy, by matching and perceptual reasoning in general, irrespective of language and formal schooling. The brief IQ composite obtained from the Leiter-R is based on four subtests: Figure Ground, Form Completion, Sequential Order, and Repeated Patterns. Similarly, the complete IQ composite obtained from the Leiter-3 is based on four subtests: Figure Ground, Form Completion, Classification and Analogies, and Sequential Order. The GMDS-ER 2–8 was administered when a child failed to complete the Leiter scales because of his/her reduced attentional resources. The GMDS-ER 2–8 was completed by 53 children, 28 children completed Griffith III, while the Leiter scales were completed by 48 children (Leiter 3 was completed by 44 children and Leiter-R scale was completed by 4 children).

### 2.3 MR imaging acquisition

All ASD patients and HCs underwent brain MR imaging in the same Institution (Bambino Gesù Children’s Hospital, Rome) on a 1.5 T (Magnetom Aera, Siemens, Erlangen, Germany) or a 3 T (Magnetom Skyra, Siemens Erlangen, Germany) scanner. Brain MRI protocol consisted of a T1-weighted 3D magnetization-prepared rapid gradient echo (MP-RAGE) sequence (TR = 2060 ms, TE = 2.27 ms, TI = 1,040 ms, FA = 9°, ST = 1 mm).

### 2.4 Image analysis

Data were pre-processed with FreeSurfer 5.3 software,^1^ using a standard automatic pipeline (i.e., recon-all) that sequentially performed skull stripping, intensity correction and transformation to Talairach-Tournoux space to produce grey matter (GM) and white matter (WM) segmentation. Specifically, motion correction was performed prior to averaging when using different source volumes in order to compensate for small variations in motion between volumes. In addition, intensity normalisation was applied to the original volume and the intensities of all voxels were scaled to the mean value of the white matter (23). After correcting the movements and normalising the data, FS removed the skull to isolate the brain from extracranial or non-brain tissue in a process known as skull stripping (24). Particularly, combining information from tissue intensity and neighborhood constraints, the FreeSurfer automatic pipeline, firstly determined and then tessellated the GM–WM boundary to generate the inner cortical surface (white surface). The outer surface (pial surface) was generated through the expansion of the white surface with a point-to-point correspondence. For each subject, the FreeSurfer automatic pipeline computed the CT parameter as the average distance measured from each surface to the other, according to Fischl and Dale approach (25). FreeSurfer-preprocessed scans quality was assessed using the Qoala-T Tool (26).^2^ Scans that had a borderline Qoala-T score were also visually inspected. The reconstructed white and pial surfaces were visually checked to verify and correct any algorithmic misinterpretation of gyri and sulci. LGI were computed vertex-wise over the entire cortex using the method of Schaer et al., which measures the amount of cortex buried within the sulcal folds as compared with the amount of visible cortex in spherical regions of interest (ROIs) (27).

### 2.5 Statistical analysis

In order to pool data from our 2 different scanners, we adjust the CT and LGI values via COMBAT-GAM approach. The CT and LGI scanner variability was removed by including diagnosis, age, and sex as biological variables, and age was specified as a non-linear term in the model. The ComBat-GAM code used was implemented in Python (ver. 3.7.6).^3^ The corrected data were then used for the further comparison analysis.

Intergroup differences were analyzed through analysis of variance. We investigated differences in cortical parameter distributions among groups. To this purpose, we mapped vertex-wise CT and LGI values on a common spherical coordinate system (i.e., fsaverage), using spherical transformation. A two-group Generalized Linear Model (GLM) analyses of both CT and LGI measures were performed by vertex-wise analysis with permutation-based cluster correction for multiple comparisons (mri_glmfit). Permutation correction was done by permuting the design matrix, recomputing the significance map, thresholding, and extracting the largest cluster over 1,000 iterations. The value of p for a cluster in the real data was then computed as the probability of seeing a maximum cluster of that size or larger in a given hemisphere, followed by the correction for two hemispheres (28). Subject age was set as a silent regressor in the group intercept GLM model. Bidirectional contrasts were applied to both CT and LGI analyses (i.e., group1 > group2, group1 < group2). This cluster-wise correction simulation (repeated over 5,000 iterations) is a way to get a measure of the maximum cluster size distribution under the null hypothesis. Resulted clusters were displayed on a common inflated surface template. All the statistical analysis were corrected for the global brain size, setting the total intracranial volume as covariate. The effect of the gender was considered adding in the model of the statistical analysis the gender as covariate.

## 3 Results

### 3.1 Subjects

Between January 2016 and December 2018, a total 140 subjects were enrolled in the study. Among 140 subjects, 129 ASD patients (20 females and 109 males, mean age = 5.17 y, age range = 2.4–8 y) underwent MRI investigation. Among ASD group, 71/135 ASD patients demonstrated verbal ability (ASD_VERB1, mean age = 5.2 y), while the remaining 58/135 patients revealed no verbal ability (ASD_VERB2; mean age = 5,1 y), operationalized as the absence of fluent language during the clinical observation. Moreover, ASD patients were also split according to the IQ score, revealing 54/135 ASD patients with IQ greater than 70 (ASD_IQ1, mean age = 5.2 y) and 75/135 with IQ less than 70 (ASD_IQ2, mean age 5.1 y). A group of 58 HC (24 females and 34 males, mean age = 4.8 y, age range = 2–8 y) was also included. The reason for MRI brain acquisition was suspected spinal dysraphism (12/58), headache (17/58), vertigo (4/58), pineal cyst (3/58), facial vascular malformation (4/58), delayed growth (3/58); precocious puberty (3/58); torticollis (3/58); strabismus (6/58) and syncope (3/58).

### 3.2 Overall results

**Figure 1:**
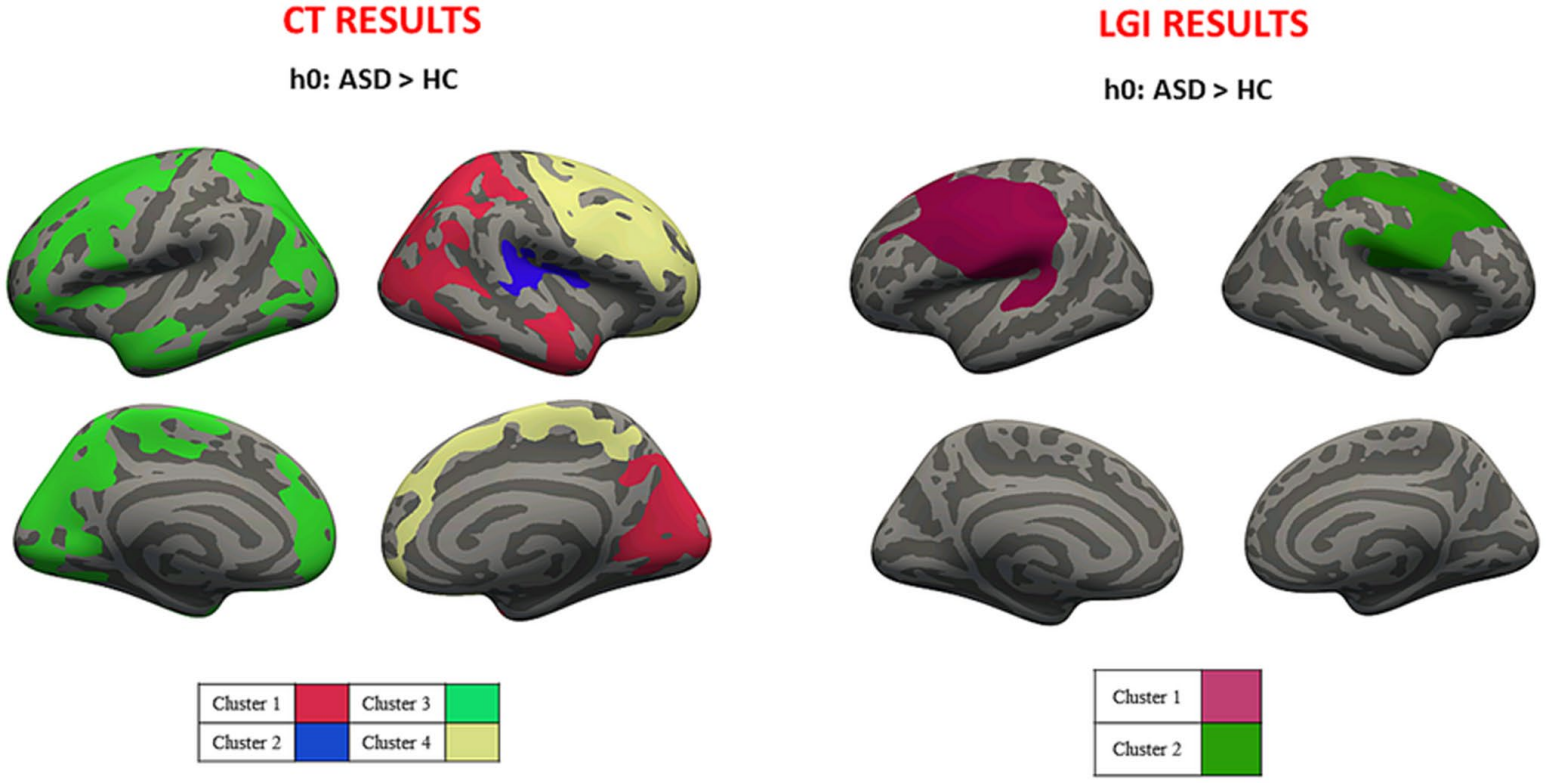
Cluster-wise analysis mapped on the inflated common surface. Overall CT (left) and LGI (right) results, where each color is associated with different clusters; CT, cortical thickness; LGI, local gyrification.

Average values of CT and LGI were computed for both hemispheres in ASD (CT_LEFT_ = 2.829 ± 0.789 mm; CT_RIGHT_ = 2.831 ± 0.789 mm; LGI_LEFT_ = 3.455 ± 0.931; LGI_RIGHT_ = 3.459 ± 0.901) and HC (CT_LEFT_ = 2.780 ± 0.778 mm; CT_RIGHT_ = 2.785 ± 0.776 mm; LGI_LEFT_ = 3.406 ± 0.807; LGI_RIGHT_ = 3.419 ± 0.862). Cluster-wise analysis revealed cortical areas of significantly higher CT and LGI values for ASD patients when compared to HC (Figure 1). Table 1 reported both CT and LGI results obtained for ASD-HC comparison, showing cortical lobe, and mean cortical parameter values of both groups for each cluster of significant results.

**Table 1:** Overall CT and LGI results for ASD-HC comparison.

G1 > G2 condition	Lateral	# clusters	Lobes	% significant lobe area	*p*-value	G1 mean CT	G2 mean CT
ASD > HC	LH	1/1	Frontal	12.6%	0.002	2.82 (0.81)	2.76 (0.80)
			Parietal	11.1%	0.002	2.84 (0.73)	2.79 (0.72)
			Temporal	9.1%	0.002	2.80 (0.84)	2.75 (0.83)
			Occipital	9.8%	0.002	2.85 (0.76)	2.80 (0.75)
	RH	1/3	Parietal	4.11%	0.002	2.88 (0.75)	2.83 (0.73)
			Temporal	8.94%	0.002	2.83 (0.80)	2.78 (0.79)
			Occipital	8.49%	0.002	2.84 (0.76)	2.79 (0.74)
		2/3	Frontal	12.85%	0.002	2.83 (0.77)	2.78 (0.76)
			Parietal	2.21%	0.002	2.79 (0.87)	2.74 (0.85)
		3/3	Frontal	2.36%	0.002	2.81 (0.85)	2.76 (0.84)

G1 > G2	Lateral	# clusters	Lobes	% significant lobe area	*p*-value	G1 mean LGI	G2 mean LGI
ASD > HC	LH	1/1	Frontal	12.19%	0.002	3.43 (0.91)	3.39 (0.87)
			Parietal	9.52%	0.002	3.41 (0.90)	3.36 (0.86)
			Temporal	6.53%	0.002	3.42 (0.94)	3.37 (0.89)
			Occipital	1.20%	0.002	3.39 (0.94)	3.34 (0.90)
	RH	1/1	Frontal	10.96%	0.002	3.44 (0.89)	3.40 (0.85)
			Parietal	7.59%	0.002	3.45 (0.91)	3.40 (0.87)
			Temporal	4.72%	0.002	3.49 (0.94)	3.44 (0.90)

The value of ps reported are corrected for multiple comparisons; CT, cortical thickness; LGI, local gyrification; ASD, autism spectrum disorder; HC, health control.

### 3.3 Verbal ability results

**Figure 2:**
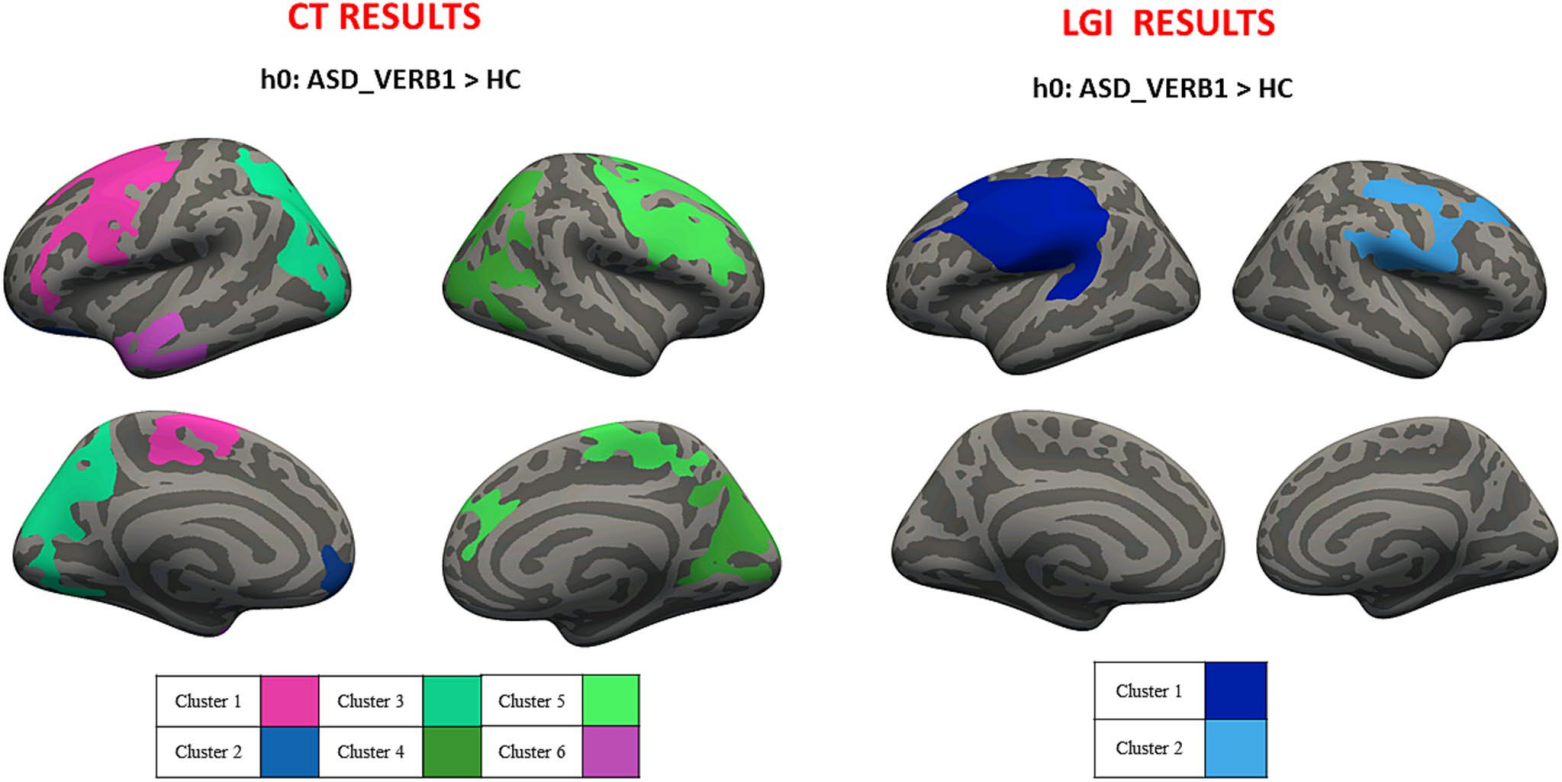
Cluster-wise analysis mapped on the inflated common surface. CT (left) and LGI (right) results for VERB1-HC comparison, where each color is associated with different clusters; CT, cortical thickness; LGI, local gyrification; VERB1, verbal ability – health control.

**Figure 3:**
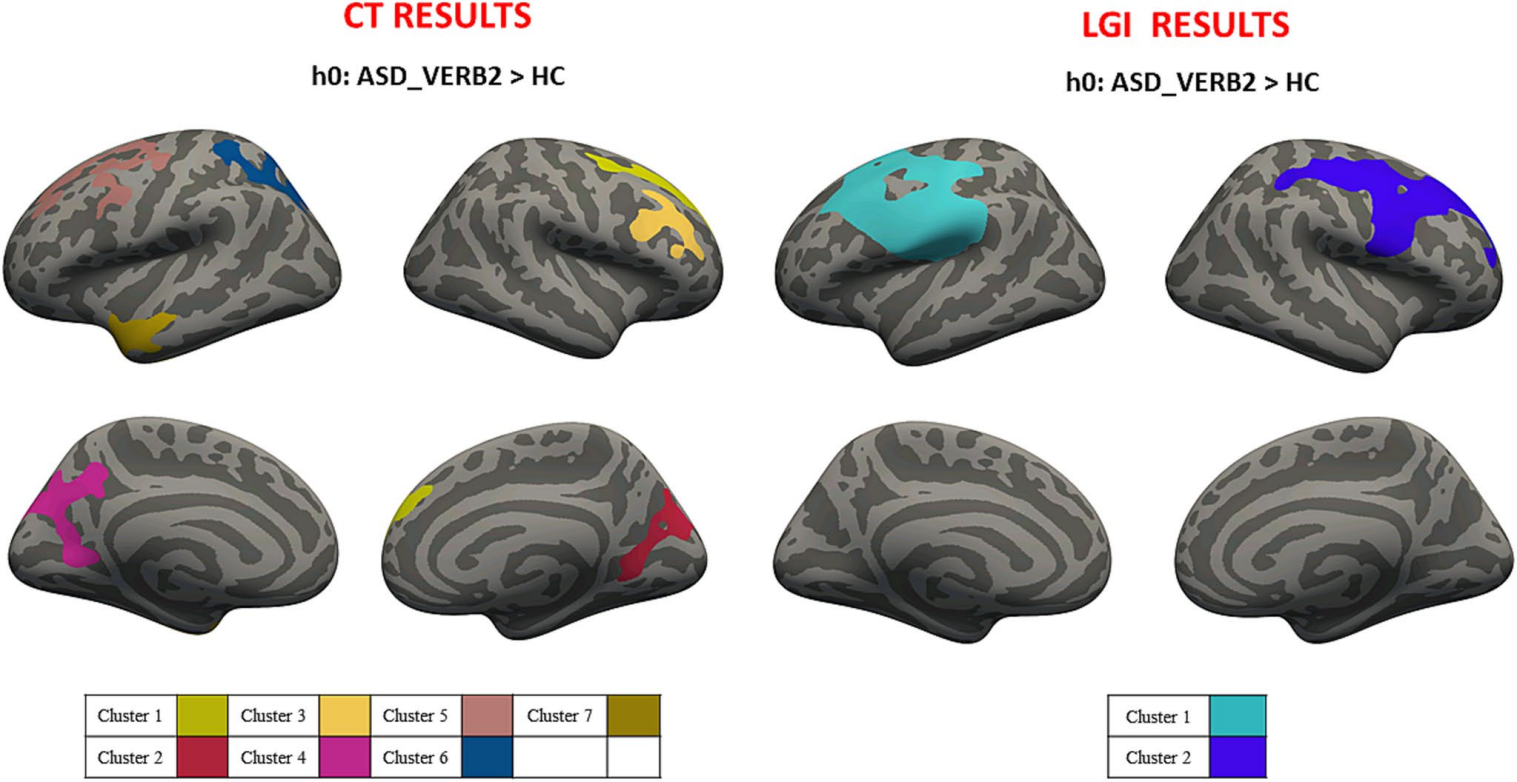
Cluster-wise analysis mapped on the inflated common surface. CT (left) and LGI (right) results for VERB2-HC comparison, where each color is associated with different clusters; CT, cortical thickness; LGI, local gyrification; VERB2, no verbal ability; HC, health control.

Average values of CT and LGI were computed for both hemispheres in ASD_VERB1 (CT_LEFT_ = 2.834 ± 0.791 mm; CT_RIGHT_ = 2.837 ± 0.793 mm; LGI_LEFT_ = 3.457 ± 0.941; LGI_RIGHT_ = 3.466 ± 0.916) and ASD_VERB2 (CT_LEFT_ = 2.823 ± 0.792 mm; CT_RIGHT_ = 2.824 ± 0.790 mm; LGI_LEFT_ = 3.452 ± 0.91; LGI_RIGHT_ = 3.450 ± 0.882). Cluster-wise analysis revealed cortical areas of significantly higher CT and LGI values for both ASD_VERB1 and ASD_VERB2 patients when compared to HC (respectively Figures 2, 3). All the statistical verbal ability results were summarized in Table 2.

**Table 2:** Language CT and LGI results for ASD subgroups (VERB1 and VERB2) and HC comparison.

G1 > G2 condition	Lateral	# clusters	Lobes	% significant lobe area	*p*-value	G1 mean CT	G2 mean CT
VERB1 > HC	LH	1/2	Frontal	8.89%	0.002	2.79 (0.83)	2.73 (0.81)
			Parietal	9.12%		2.85 (0.73)	2.79 (0.72)
			Temporal	5.39%		2.80 (0.84)	2. 74 (0.83)
			Occipital	7.26%		2.85 (0.78)	2.79 (0.76)
		2/2	Frontal	1.41%	0.028	2.84 (0.81)	2.79 (0.79)
	RH	1/3	Parietal	3.43%	0.002	2.90 (0.75)	2.85 (0.74)
			Occipital	6.86%		2.84 (0.76)	2.78 (0.74)
		2/3	Frontal	11.13%	0.002	2.83 (0.78)	2.78 (0.77)
		3/3	Temporal	6.38%	0.002	2.81 (0.83)	2.76 (0.81)

G1 > G2	Lateral	# clusters	Lobes	% significant lobe area	*p*-value	G1 mean LGI	G2 mean LGI
VERB1 > HC	LH	1/1	Frontal	12.55%	0.002	3.46 (0.93)	3.41 (0.88)
			Parietal	9.71%		3.41 (0.92)	3.36 (0.87)
			Temporal	8.59%		3.43 (0.95)	3.37 (0.90)
			Occipital	1.16%		3.40 (0.96)	3.36 (0.91)
	RH	1/1	Frontal	9.02%	0.002	3.46 (0.91)	3.42 (0.86)
			Parietal	7.24%		3.47 (0.93)	3.42 (0.88)
			Temporal	9.36%		3.49 (0.94)	3.44 (0.88)
			Occipital	3.36%		3.56 (1.00)	3.51 (0.94)

G1 > G2	Lateral	# clusters	Lobes	% significant lobe area	*p*-value	G1 mean CT	G2 mean CT
VERB2 > HC	LH	1/5	Parietal	1.02%	0.002	2.75 (0.79)	2.71 (0.77)
			Temporal	5.11%		2.77 (0.86)	2.72 (0.85)
		2/5	Occipital	1.74%	0.002	2.81 (0.83)	2.77 (0.81)
		3/5	Parietal	1.34%	0.004	2.85 (0.69)	2.80 (0.67)
		4/5	Temporal	1.05%	0.004	2.89 (0.81)	2.85 (0.82)
		5/5	Parietal	1.20%	0.049	2.88 (0.66)	2.84 (0.66)
	RH	1/5	Temporal	3.70%	0.002	2.79 (0.84)	2.75 (0.83)
		2/5	Frontal	2.16%	0.002	2.81 (0.76)	2.76 (0.74)
		3/5	Frontal	1.57%	0.018	2.92 (0.58)	2.88 (0.57)
		4/5	Occipital	0.85%	0.030	2.93 (0.68)	2.89 (0.67)
		5/5	Temporal	1.12%	0.032	2.87 (0.76)	2.82 (0.73)

G1 > G2	Lateral	# clusters	Lobes	% significant lobe area	*p*-value	G1 mean LGI	G2 mean LGI
VERB2 > HC	LH	1/1	Frontal	13.47%	0.002	3.41 (0.90)	3.37 (0.87)
			Parietal	7.00%		3.41 (0.89)	3.36 (0.86)
			Limbic	1.06%		3.31 (0.84)	3.27 (0.81)
			Temporal	2.42%		2.43 (0.88)	3.39 (0.85)
	RH	1/1	Frontal	8.89%	0.002	3.42 (0.87)	3.39 (0.85)
			Parietal	3.36%		3.50 (0.88)	3.46 (0.87)

G1 > G2	Lateral	# clusters	Lobes	% significant lobe area	*p*-value	G1 mean CT	G2 mean CT
VERB1 > VERB2	LH	0	–	–	–	–	–
	RH	1/1	Occipital	1.10%	0.006	2.89 (0.66)	2.87 (0.65)

G1 > G2	Lateral	# clusters	Lobes	% significant lobe area	*p*-value	G1 mean LGI	G2 mean LGI
VERB1 > VERB2	LH	0	–	–	–	–	–
	RH	1/1	Temporal	2.87%	0.024	3.53 (0.94)	3.52 (0.90)
			occipital	2.42%		3.55 (1.02)	3.53 (0.99)

The *p*-values reported are corrected for multiple comparisons; CT, cortical thickness; LGI, local gyrification; ASD, autism spectrum disorder; VERB1, verbal ability; VERB2, no verbal ability; HC, health control.

### 3.4 IQ results

**Figure 4:**
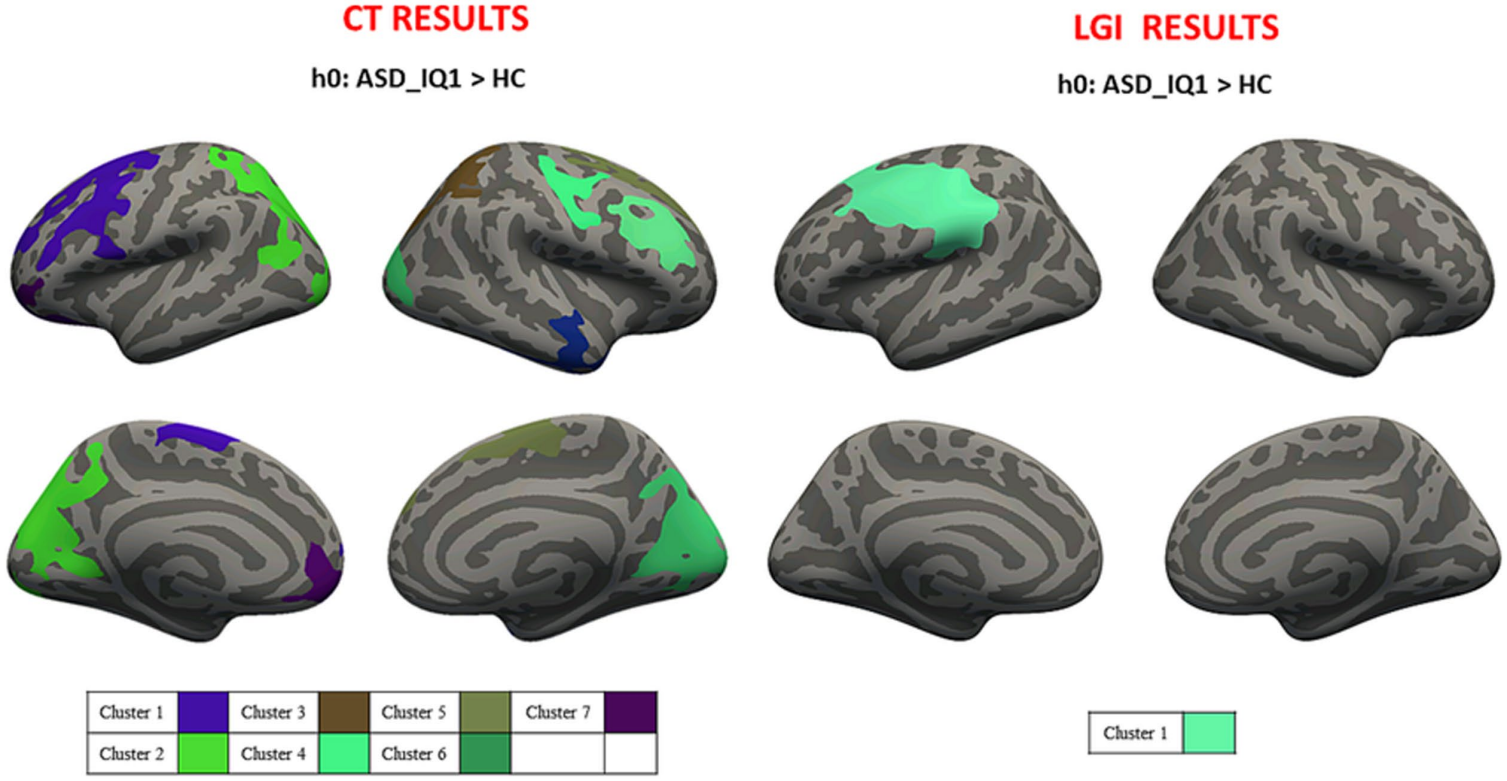
Cluster-wise analysis mapped on the inflated common surface. CT (left) and LGI (right) results for IQ1-HC comparison, where each color is associated with different clusters; CT, cortical thickness; LGI, local gyrification; IQ1, intelligence quotient>70; HC, health control.

**Figure 5:**
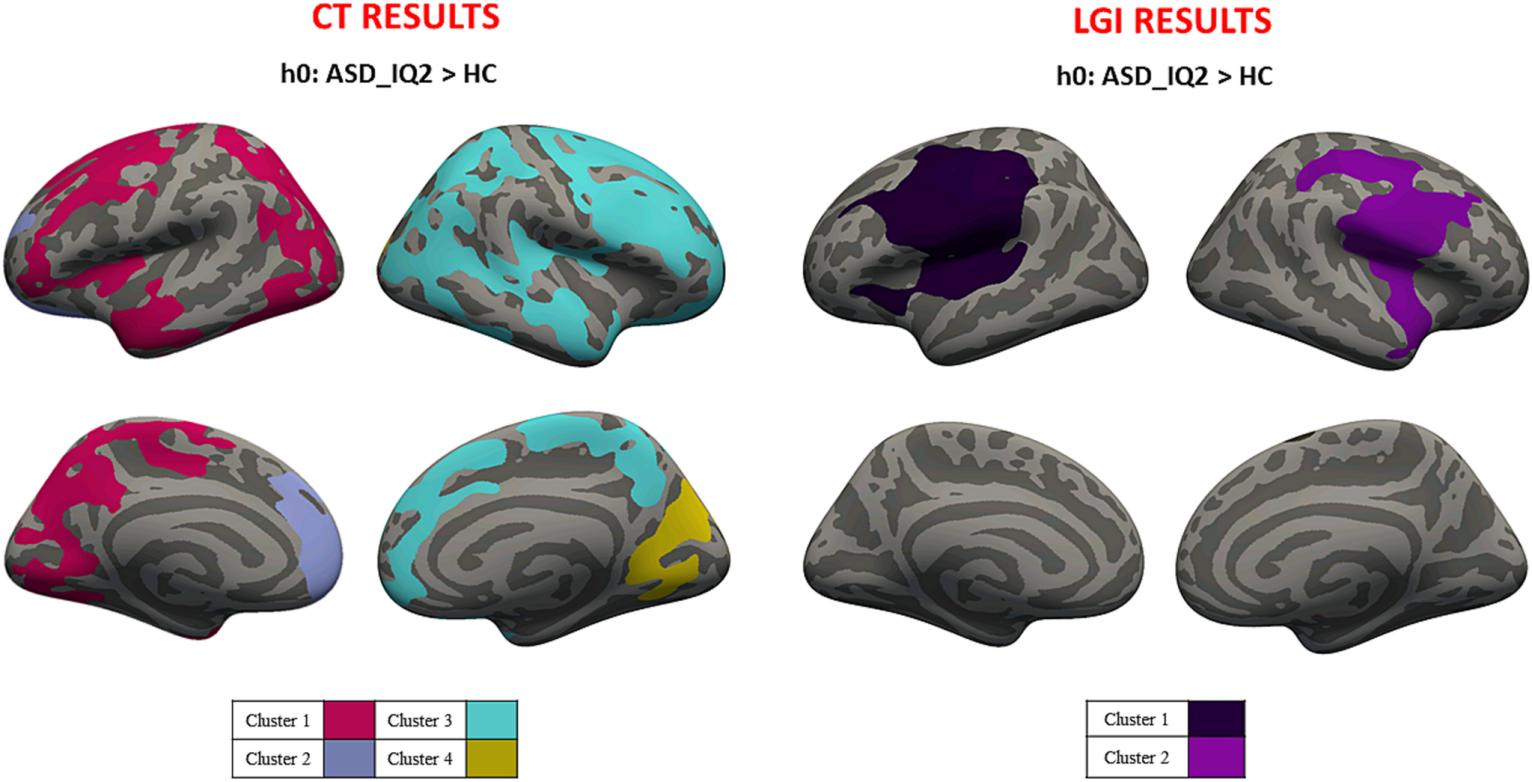
Cluster-wise analysis mapped on the inflated common surface. CT (left) and LGI (right) results for IQ2-HC comparison, where each color is associated with different clusters; CT, cortical thickness; LGI, local gyrification; IQ2, intelligence quotient<70; HC, health control.

Average values of CT and LGI were computed for both hemispheres in ASD_IQ1 (CT_LEFT_ = 2.823 ± 0.786 mm; CT_RIGHT_ = 2.815 ± 0.786 mm; LGI_LEFT_ = 3.440 ± 0.926; LGI_RIGHT_ = 3.443 ± 0.892) and ASD_IQ2 (CT_LEFT_ = 2.833 ± 0.795 mm; CT_RIGHT_ = 2.843 ± 0.796 mm; LGI_LEFT_ = 3.466 ± 0.935; LGI_RIGHT_ = 3.471 ± 0.908). Cluster-wise analysis revealed cortical areas of significantly higher CT and LGI values for both ASD_IQ1 and ASD_IQ2 patients when compared to HC (respectively Figures 4, 5). The ASD_IQ1 group also revealed cortical areas of decreased CT within the limbic lobe of right hemisphere when compared to HC. No significant results were found when comparing CT and LGI between ASD_IQ1 and ASD_IQ2. All the statistical IQ results were summarized in Table 3.

**Table 3:** IQ CT and LGI results for ASD subgroups (IQ1 and IQ2) and HC comparison.

G1 > G2 condition	Lateral	# clusters	Lobes	% significant lobe area	*p*-value	G1 mean CT	G2 mean CT
IQ1 > HC	LH	1/3	Parietal	5.92%	0.002	2.86 (0.68)	2.81 (0.68)
			Temporal	4.62%		2.79 (0.83)	2. 74 (0.82)
			Occipital	7.67%		2.83 (0.77)	2.78 (0.75)
		2/3	Frontal	5.97%	0.002	2.77 (0.85)	2.73 (0.84)
			Parietal	1.62%		2.84 (0.75)	2.78 (0.74)
		3/3	Frontal	1.45%	0.014	2.84 (0.79)	2.80 (0.76)
	RH	1/6	Occipital	4.73%	0.002	2.82 (0.76)	2.79 (0.75)
		2/6	Frontal	1.74%	0.006	2.87 (0.63)	2.83 (0.62)
		3/6	Frontal	2.10%	0.006	2.69 (0.93)	2.66 (0.92)
		4/6	Occipital	1.06%	0.008	2.97 (0.57)	2.94 (0.55)
		5/6	Frontal	1.79%	0.008	2.82 (0.76)	2.79 (0.75)
		6/6	Temporal	2.42%	0.01	2.85 (0.80)	2.82 (0.79)

G1 > G2 condition	Lateral	# clusters	Lobes	% significant lobe area	*p*-value	G1 mean CT	G2 mean CT
HC > IQ1	LH	0	–	–	–	–	–
	RH	1/1	Limbic	1.37%	0.002	2.78 (0.81)	2.74 (0.80)

G1 > G2	Lateral	# clusters	Lobes	% significant lobe area	*p*-value	G1 mean LGI	G2 mean LGI
IQ1 > HC	LH	1/1	Frontal	6.70%	0.002	3.40 (0.91)	3.37 (0.87)
			Parietal	6.60%		3.39 (0.89)	3.37 (0.86)
			Temporal	1.67%		3.43 (0.89)	3.39 (0.85)
	RH	1/1	Frontal	5.19%	0.002	3.41 (0.88)	3.39 (0.85)
			Parietal	2.06%		3.54 (0.91)	3.51 (0.88)

G1 > G2	Lateral	# clusters	Lobes	% significant lobe area	*p*-value	G1 mean CT	G2 mean CT
IQ2 > HC	LH	1/5	Frontal	1.87%	0.002	2.89 (0.54)	2.85 (0.65)
			Parietal	7.69%		2.87 (0.56)	2.79 (0.72)
			Temporal	1.52%		2.86 (0.56)	2.85 (0.74)
			Occipital	8.26%		2.85 (0.58)	2.79 (0.77)
		2/5	Parietal	1.65%	0.002	2.81 (0.60)	2.72 (0.77)
			Temporal	6.38%		2.85 (0.57)	2.73 (0.83)
		3/5	Frontal	3.86%	0.002	2.81 (0.59)	2.74 (0.84)
		4/5	Frontal	2.84%	0.002	2.85 (0.55)	2.76 (0.82)
		5/5	Frontal	1.40%	0.01	2.78 (0.62)	2.70 (0.82)
	RH	1/2	Frontal	13.92%	0.002	2.86 (0.76)	2.80 (0.74)
			Parietal	8.75%		2.87 (0.77)	2.81 (0.76)
			Temporal	8.86%		2.82 (0.84)	2.76 (0.82)
			Occipital	6.74%		2.87 (0.75)	2.81 (0.73)
		2/2	Frontal	2.37%	0.002	2.82 (0.83)	2.77 (0.82)

G1 > G2	Lateral	# clusters	Lobes	% significant lobe area	*p*-value	G1 mean LGI	G2 mean LGI
IQ2 > HC	LH	1/1	Frontal	14.82%	0.002	3.44 (0.93)	3.39 (0.88)
			Parietal	10.00%		3.41 (0.90)	3.36 (0.86)
			Temporal	8.55%		3.44 (0.94)	3.38 (0.89)
			Occipital	1.37%		3.37 (0.94)	3.31 (0.90)
	RH	1/1	Frontal	11.51%	0.002	3.44 (0.91)	3.39 (0.86)
			Parietal	7.36%		3.55 (0.91)	3.40 (0.86)
			Temporal	5.71%		3.48 (0.92)	3.42 (0.88)

The *p*-values reported are corrected for multiple comparisons; CT, cortical thickness; LGI, local gyrification; ASD, autism spectrum disorder; IQ1, intelligence quotient>70; IQ2, intelligence quotient<70; HC, health control.

## 4 Discussion

The first aim of this study was to compare measures of cortical thickness and local gyrification between a large sample of preschoolers and schoolers ASD and a group of neurotypical children matched for gender and age. The results of our study showed abnormalities in LGI and CT in patients affected by ASD compared to HC and among the subgroups of patients. This evidence suggests that ASD is a complex neurodevelopmental disorder dependent on brain abnormalities presenting from a very young age, probably during a prenatal life and the first three postnatal years (29).

### 4.1 Cortical thickness

Inconsistencies result from several neuroimaging studies of cortical morphometry, showing both increased, decreased CT, and no CT differences in ASD compared to HC (12, 30–33). These evidences could be related to differences in diagnostic/inclusion criteria, age, patient characteristics (e.g., intelligence quotient), heterogeneity of the disorder and the small sample size considered (34, 35). Additionally, neuroimaging studies on ASD patients have been reported inconsistent findings across factors influencing clinical heterogeneity and their relationship to brain anatomy. In this study, morphological characteristic in ASD subgroups was also evaluated, distinguishing ASD in terms of clinical subtypes based on IQ and verbal abilities in order to evaluate the relationship between brain morphometry and different clinical phenotypes. When compared to HC, both the complete ASD group and the subgroups (i.e., ASD_VERB1, ASD_VERB2, ASD_IQ1 and ASD_IQ2) revealed a significant CT increase over broad cortical areas of both hemispheres. In particular, we found CT abnormalities in inferior frontal cortex, superior temporal sulcus, cingulate gyrus, middle occipital gyrus, fusiform gyrus, and inferior parietal lobule, that cooperate in socially-relevant brain processing (36). Additionally, abnormalities found in orbital frontal gyrus, and anterior cingulate gyrus could be related to the deficit in repetitive behaviors execution (37). An increased CT in inferior frontal gyrus, superior temporal sulcus, inferior parietal lobule may play a role in non-verbal communicative behaviors (32), and a CT increased in pre- and postcentral gyri may influence facial reactions (38). These results are consistent with recent studies showing that developmental patterns of CT abnormalities reflect delayed cortical maturation, emphasizing the dynamic nature of morphological abnormalities in ASD (12, 39, 40). Particularly Hardan et al. found increasing CT in young ASD both in cerebrum and several lobes including frontal, parietal, temporal and occipital (12). Additionally, Khundrakpam and colleagues observed increased CT in children with ASD versus HC in several cortical regions from 6 years onwards until about 20 years. Since CT MRI-based measures are based on the placement of *white* and *pial* surfaces on the MRI image, the increased CT in children with ASD underlined with MRI is likely related to differences in both GM and WM (40). CT increase found in ASD could be related to microstructural changes in GM including larger numbers of neurons or glia, greater dendritic arborization, more synapses, larger or more axons, or greater capillary support (41–43) and to differences in WM, reflecting reductions in the degree of myelination, the number of myelinated axons or a relative increase in myelin adjacent to the cortex (40). These findings are also supported by the study of Hyde et al. that found increased CT in several brain areas when comparing ASD with verbal ability to HC (44). Particularly we found a CT increase in superior temporal sulcus and inferior frontal gyrus in both hemispheres that could be related to the deficit in communication (36). The clinical heterogeneous phenotypes of ASD involve also the ability of language, which may range from typical onset and development of language to difficulties in speech and language and in the absence of verbal abilities (45). Sharda et al. (46) supported the role of CT as a functional biomarker for language abilities in children with ASD founding a more severe involvement of the frontal regions in patients with more compromised verbal abilities. Our results revealed a different number of cortical areas with significant CT increase when comparing both ASD_VERB1 and ASD_VERB2 to HC. When ASD_QI1 patients were compared to HC, CT increases were observed in areas in both hemispheres, while significant reductions were observed in posterior-cingulate, caudal-anterior-cingulate and rostra-anterior-cingulate in right hemisphere. Similarly, when ASD_QI2 were compared to HC, CT increases were observed in several area. These CT differences were greater in individuals with lower IQ. Our results are consistent with those of Bedford et al. (39), that found greater CT (and greater in individual with lower IQ) in regions including the superior cortical gyrus and inferior frontal sulcus. Additionally, according to our study Hyde et al. revealed increased CT in ASD with average intelligence (47).

### 4.2 Local gyrification index

We demonstrated a significant LGI increase in ASD children compared to HC. In particular, we found a LGI increase in several cortical areas including bilateral fronto-insular regions, thus supporting evidences from previous work. Kohli et al. found an increase of LGI in left parietal and temporal regions and in right frontal and temporal regions in ASD subjects compared to HC, with a trend of bilateral reduction of LGI with age, more steeply in ASD in left precentral, right lateral occipital, and middle frontal region (14). Although we did not find any laterality when comparing ASD patients to HC, a laterality trend occurred when analyzing the patient subgroups (i.e., verbal ability and IQ). The hemispheric gyrification differences observed could reflect a wide range of presentation of ASD, including individual characteristics such as intelligence quotient as well as verbal abilities. In this context, Duret et al. found lower gyrification in a fusiform visual area in ASD subjects with speech onset delay, whereas gyrification increase occurred in a temporal language-related region in ASD without speech onset delay, thus suggesting that regional gyrification differences may reflect different cognitive defects in subjects with ASD (48). In conclusion, these results support the hypothesis of abnormal brain maturation in ASD since early childhood with differences among clinical subgroups suggesting different anatomical substrates underlying an aberrant connectivity.

## 5 Limitations

The MRI data in this study were analyzed with FreeSurfer version 5.3. This leads to limitations since a newer version of Freesurfer (i.e., version 6, 7), certainly might provide more robust results (49). However, the differences in robustness between versions is not huge, and we believe that visual inspection and subsequent manual correction mitigated the problem.

## 6 Conclusion

Our results support the hypothesis of abnormal brain maturation in ASD since early childhood with differences among clinical subgroups suggesting different anatomical substrates underlying an aberrant connectivity. Some limitations should be considered when interpreting the results of the current study. They include the cross-sectional research design. Longitudinal studies would be indicated to test morphologic alterations in ASD brain maturation clarifying how the trajectories change in following age. Second the sample studied comprises a large number of participants limited to patients attending the same Tertiary Care Hospital and with a underrepresented group of female. Finally verbal abilities have not been quantified by specific speech assessment. Future research should aim to fill research gaps by addressing multiple issues, such as exploring brain maturation in large groups of ASD in longitudinal studies.

## Data availability statement

The raw data supporting the conclusions of this article will be made available by the authors, without undue reservation.

## Ethics statement

The studies involving humans were approved by Ospedale Pediatrico Bambino Gesù. The studies were conducted in accordance with the local legislation and institutional requirements. Written informed consent for participation in this study was provided by the participants’ legal guardians/next of kin.

## Author contributions

AN, SG, and SV designed the experiments. ME, LT, and SG carried out the experiments. AN, ML, and CP analyzed the experimental results. AN, SG, and ML wrote the manuscript. SG, CP, and GB reviewed and edited the manuscript. All authors contributed to the article and approved the submitted version.

## Footnotes

^1^ http://surfer.nmr.harvard.edu/

^2^ https://github.com/Qoala-T/Qoala-T

^3^ https://github.com/rpomponio/neuroHarmonize
